# But it’s legal, isn’t it? Law and ethics in nursing practice related to medical assistance in dying

**DOI:** 10.1111/nup.12277

**Published:** 2019-08-19

**Authors:** Catharine J. Schiller, Barbara Pesut, Josette Roussel, Madeleine Greig

**Affiliations:** ^1^ University of Northern British Columbia Prince George BC Canada; ^2^ University of British Columbia Okanagan, Kelowna BC Canada; ^3^ Canadian Nurses Association Ottawa ON Canada

**Keywords:** ethics, euthanasia, legal, medical assistance in dying, moral

## Abstract

In June 2015, the Supreme Court of Canada struck down the *Criminal Code's* prohibition on assisted death. Just over a year later, the federal government crafted legislation to entrench medical assistance in dying (MAiD), the term used in Canada in place of physician‐assisted death. Notably, Canada became the first country to allow nurse practitioners to act as assessors and providers, a result of a strong lobby by the Canadian Nurses Association. However, a legislated approach to assisted death has proven challenging in a number of areas. Although it facilitates a degree of accountability, precision and accessibility, it has also resulted in particular challenges negotiating the diverse perspectives of such a morally contentious act. One of these challenges is the tendency to conflate what is legal and what is moral in a modern liberal constitutionalism that places supreme value on autonomy and choice. Such a conflation tends to render invisible the legal and moral/ethical considerations necessary for nurses and nurse practitioners to remain ethical actors. In this paper, we introduce this conflation and then discuss the process of lawmaking in Canada, including the legalization of MAiD and the contributions of nursing to that legalization. We then engage in a hypothetical dialogue about the legal and moral/ethical implications of MAiD for nursing in Canada. We conclude with an appeal for morally sustainable workspaces that, when implementing MAiD, appropriately balance patient choices and nurses’ moral well‐being.

## 1 INTRODUCTION

The legalization of medical assistance in dying (MAiD) in Canada has had significant implications for Canadian nursing. Canada is the first country to allow nurse practitioners (NPs)1Nurse Practitioners in Canada are advanced practice nurses, often educated at the master's level, who have the authority to independently diagnose and prescribe for a range of health conditions. to act as MAiD assessors and providers (i.e. assess patients for their eligibility for MAiD and either administer or prescribe the medications leading to death). Registered nurses in Canada also have important supportive roles in coordinating and providing wrap‐around care for MAiD. The complexities of this new end‐of‐life option have resulted in steep learning curves for all those involved; these lessons may benefit nurses in other countries where the implementation of assisted death is being considered or illuminate unknown challenges in jurisdictions where assisted death is already allowed. The purpose of this paper is to discuss some of the nursing practice implications of a legislated approach to assisted death and, in particular, to show the potentially complex interplay between law and morality/ethics regarding this act.

This question of the interplay of law and morality/ethics first came to our attention when we were interviewing Canadian nurses about their experiences with MAiD (Pesut, Thorne, Schiller, Greig, & Roussel, 2019). The nurses who participated in that study sometimes resolved their moral uncertainty about MAiD by appealing to its legality. For example, a participant would begin to speak of their discomfort with MAiD but then conclude with, “but it's legal”. Such comments suggest the nurses may have been conflating, or at least not clearly differentiating between, their legal and moral/ethical commitments; alternatively, they may not have realized that differing legal and moral/ethical commitments could lead them to a different conclusion about their participation in MAiD.

This conflation is characteristic of liberal societies in which political ideologies tend to homogenize notions of the good. For example, Deneen (2018) has argued that the current modern liberal constitutionalism is based largely upon anthropological assumptions of individualism and choice. Liberty, defined as individual freedom of choice, then becomes the criterion by which we judge the goodness of a society's law, policy and morality. This idea was echoed in our data as nurses suggested that their primary reason for engaging with MAiD was to support patient autonomy and choice, a primary good that shaped nurses’ perceptions of their legal and ethical obligations. However, not all nurses gave primacy to these values of choice and autonomy. For example, some nurses declared themselves to be conscientious objectors based upon other principles such as communalism or an unwillingness to end a life. A final group of nurses were less certain. They acknowledged the legality of MAiD, participated in it to varying degrees, but then found themselves experiencing a moral uncertainty they had difficulty interpreting.

Participants who existed within that grey zone of legal certainty but moral uncertainty pushed us, as investigators, to consider how nurses think about the interplay of law and morality/ethics. Perhaps more importantly, it required us to explore how healthcare policies that provide the context for nurses’ work environments may either support or impede nurses’ abilities to reflect and act upon both their legal and their ethical commitments. As we pondered this question, we had an illuminating moment about our own different assumptions within the group. In discussing the role of law and ethics, *CS*, a lawyer, asserted that law is the foundation of a society. *BP*, the *aspiring* ethicist, replied that's not so; instead, morality and ethics are the foundation of a society and one cannot build a system of justice without them. Clearly, we were revealing our own assumptions about the ways in which law and morality/ethics play out in, and affect, public life.

These ponderings about the degrees of influence of law and ethics on morally contentious nursing practices were what led to the idea for this paper. We begin from the assumption that, although law, policy and ethics together inextricably shape good nursing practice, the conflation of law and ethics also brings unique challenges into already contested spaces, such as those surrounding the practice of MAiD. We will begin by providing some key definitions and discussing the process of law development in Canada. We will then describe the process by which MAiD became law in Canada and discuss the contribution of nursing to that legislation. Finally, we will discuss the various implications of MAiD for nurses and NPs from legal and moral/ethical perspectives using a hypothetical dialogue.

## 2 KEY DEFINITIONS

It is important to clearly distinguish between law, ethics and morality. Unfortunately, these three terms are often used interchangeably by healthcare providers as well as the general public; this can lead to confusion about both the source of concern under discussion and the best approach to resolving the societal issue being debated (Jackson, 2015; Ray, 1996). With respect to MAiD specifically, issues that have been raised since its legalization tends to suggest a concern that, for many groups, the letter of the law may not entirely coincide with many of the ethical and moral positions these groups have taken. It is therefore critical that any discussion of such issues begin with a clear understanding of these terms and their influence on one another.

*Morals*: Whether or not an action is moral appeals to one's sense of the rightness or goodness of that conduct (Biggs, 2017). Morals are held to be of a universal nature and typically do not require detailed examination or questioning to be understood as the “right” thing to do (Ray, 1996). In other words, morals are ‘social conventions about right and wrong human conduct that are so widely shared that they form a stable…communal consensus’ (Beauchamp & Childress, 1994, as cited in Horner, 2003, p. 264). One example of such a moral is that we should not kill others except in exceptional circumstances such as war. If we are able to go about our lives in accordance with such moral values, we become positioned to live with purpose and integrity, bring out the good in others, and develop a community in which we all can flourish (Horner, 2003). The implementation of MAiD in Canada has been particularly challenging from a morality perspective because there is no communal consensus about the rightness of this act.

*Ethics*: Two kinds of ethics are of interest to nurses and the nursing profession: personal ethics and professional ethics. Personal ethics, in contrast to morals, are highly individualistic and are deeply rooted in acting towards the good of the individual rather than for all of society (Guido, 2014). As they are so individualized, any two people may hold a very different perspective on an ethical issue or they may share distinct similarities in their position (Keatings & Smith, 2010). Because two people can hold different yet equally defensible ethical positions on a matter, any examination or discussion of one's own personal ethics requires an acknowledgement that we could be wrong; repeatedly questioning and challenging one's own ethical position on a matter will therefore be necessary (Pasztor, 2015). This common ground and challenge has been evident in MAiD discussions when nurses agree on the need to resolve suffering for the good of the individual but then disagree on the ethical means acceptable to relieve that suffering.

Professional ethics represent the common ethics of a particular group of people and are meant to guide that profession's relationships with other people, other organizations and other professions (Pettifor, 2010). Today, most professions develop a written code or statement of ethics that members are expected to follow, and these documents can be useful to members in understanding the group's ethical course of professional action (Foster & Miola, 2015). Depending upon the particular profession, some codes may adopt a moralistic or philosophical tone while others reflect more practical and specific guidance for members (Ray, 1996). For example, the Canadian Nurses Association's ([CNA], 2017a) Code of Ethics specifies values and responsibilities for which one is accountable as a professional nurse.

*Laws*: The legal system of a given jurisdiction is defined by the government of that jurisdiction and is interpreted by its courts and regulatory authorities. The laws that form the backbone of such a system are created and enforced for the good of the broader society, even if it means that individuals may be limited in their freedoms or have obligations imposed upon them (Horner, 2003). The law sets out the minimum acceptable standards for the behaviour of societal members and, in contrast to morals and ethics, must be followed. If a person or organization does not meet the legal requirements, liabilities and punishments can result (Duthie, Jiwani, & Steele, 2017; Foster & Miola, 2015; Olick, 2001; Sokol, 2008).

While it is often assumed that the laws of a society will conform to both the morals that underpin it and the ethics of the majority of citizens within that society, that is not always the case. Certainly, law can be considered a partial barometer of what that particular society values. However, in a society where there is diversity of individuals or groups, there will often be different (even conflicting) beliefs and values at play in a given situation and it will simply not be possible for the law to reflect what is important to everyone (Duthie et al., 2017).

In addition, moral situations will often arise before laws exist to adequately deal with them (Dolan, 2017). This may occur for several reasons. First, a society that once viewed a moral dilemma in a particular way may, over time, find itself prepared to view it differently; this triggers the need for a responsive law to be created, or for an out‐of‐date law to be amended, to reflect the new position. This was the situation encountered in Canada with respect to MAiD; the original *Criminal Code* prohibition of all forms of assisted suicide needed to be revisited because the morality of Canadian society had evolved over time and a change in law was being demanded. Second, the progress of technology usually outpaces the slower speed of legal development. For example, advances in telehealth technology that enable healthcare providers to monitor and care for a person a significant distance away were not even contemplated just a few decades ago. Therefore, the privacy and confidentiality considerations associated with using telehealth technology were not reflected in court decisions or the laws at that time. The science came first, and the law needed time to begin to catch up. In both of these examples, the law was simply not prepared to be the primary source of guidance and direction for a period of time and found itself out of step with changing societal values (Keatings & Smith, 2010).

## 3 PROCESS OF LAWMAKING IN CANADA: INPUT POINTS FOR NURSING

There are a number of stages through which an idea must pass before it becomes law in Canada. Typically, federal laws begin their journey in the House of Commons (H of C), a body comprised of elected Members of Parliament (MPs) from across the country (less commonly, the bill will begin its development in the Senate). When initiated in the H of C, the bill (or draft law) is first read in that chamber; this first reading is largely a formality as there is no debate at this stage. The second reading stage is where the bill is debated in principle in the H of C. If the bill passes a vote at this stage, it is sent to a Committee of the H of C where it is studied in detail. This forms a significant opportunity for those who hold an interest in the bill to air their concerns before the Committee, and to help Committee members understand the impact the bill is likely to have. Once the Committee finishes its clause‐by‐clause examination of the bill, and proposes any amendments, the bill is returned to the H of C for its third reading whereupon it is debated and voted upon by the entire House. After passing third reading, the bill undergoes the same process in the Senate (which consists of members appointed by a Prime Minister). If the bill as is survives the third reading in the Senate, it is given Royal Assent and becomes law. If the bill has instead been amended during the Senate process, the amendments must return to the H of C and pass its vote before it can be given Royal Assent (Minister of Public Works & Government Services Canada, 2016).

As is evident from that process, there are multiple opportunities for professions such as nursing to make their voices heard and to impact the passage or content of federal legislation (Inouye, Leners, & Miyamoto, 2019). For example, nurses can approach individual MPs well before a bill is even drafted and suggest that a law on a particular issue needs to be viewed as a priority. Communication with an MP can also occur later in the process if a nurse wishes to express support or concern for a bill that is already under consideration by the government. Broader submissions can also be advanced on behalf of the nursing profession by provincial/territorial or national regulatory bodies and associations at various stages in legislation development.

Nurses have not traditionally been very active in political advocacy, the political process or health policy development (Ellenbecker et al., 2017; Patton, Zalon, & Ludwick, 2019). This historical hesitation to become politically active means that nurses have not always seen their unique healthcare vantage points and professional ethical positions act as key influences in legislative decision‐making (Grinspun, 2006; Inouye et al., 2019; O'Rourke, Crawford, Morris, & Pulcini, 2017). However, this hesitation was not the case with the legalization of MAiD in Canada.

## 4 THE LEGALIZATION OF MAID IN CANADA AND THE NURSING VOICE

A criminal prohibition on all forms of assisted suicide was in effect in Canada until 2015 when the Supreme Court of Canada (SCC) released its *Carter v Canada (Attorney General)* (“*Carter”*) decision*.* The SCC struck down the *Criminal Code's* prohibition on physician‐assisted suicide in the *Carter* ruling, deciding that the prohibition violated the Canadian *Charter of Rights and Freedoms* (“*Charter”*), but only for competent adults who were in specific clinical circumstances. However, the SCC instituted a 12‐month suspension of its declaration of invalidity to give Parliament time to craft a legislative framework for physician‐assisted suicide, recognizing that “complex regulatory regimes are better created by Parliament than by the courts” (*Carter*, 2015, para. 125). The following year, the Canadian federal government released *An Act to amend the Criminal Code and to make related amendments to other Acts (medical assistance in dying)* in response to the *Carter* decision, thereby crafting a legislative framework to govern assisted suicide across the country. Even though it has been law for over three years now, this statute is still commonly known as *Bill C‐14.*

Within *Bill C‐14*, the federal government introduced a new concept, “medical assistance in dying”, rather than using the more familiar terms of “physician‐assisted suicide” or “physician‐assisted dying”. This new language was highly purposeful; it represented a clear recognition of the range of healthcare providers who typically engage in key roles during this complex procedure, such as NPs, physicians, registered nurses and pharmacists. The legislation also gave key responsibilities in MAiD, that of assessors and providers, to NPs rather than reserving those roles solely for physicians. Once *Bill C‐14* was passed in June 2016, provincial and territorial governments and healthcare professional regulatory bodies became responsible for enacting the policies, procedures and processes that would guide MAiD‐related healthcare practice within Canada.

During this legalization process, multiple groups representing professional nursing, such as the Canadian Nurses Protective Society and the Canadian Nurses Association, submitted briefs and presented to the Committee about their concerns with wording, practical impact and/or philosophical positions evident within the Bill ([Link]). For example, the CNA (2015) argued strongly for nursing involvement, and in particular, for the right of NPs to act as assessors and providers. The CNA had long been monitoring and discussing the implications of MAiD on nursing practice. Their advocacy work significantly increased in 2015 as the SCC, and the Quebec Court of Appeal released key decisions on MAiD. In October 2015, the CNA provided its views before the federal government's External Panel on Options for a Legislative Response to *Carter v. Canada.* The panel included key parliamentarians and bureaucrats working in the federal departments of health and justice. In March and April 2016, the CNA followed this panel presentation by meeting with many other government officials and parliamentarians.

The CNA’s goal had consistently been to recognize the importance of nurses and other members of the interprofessional healthcare team in MAiD. Nurses support patients and families during end‐of‐life care planning and act as vocal advocates for improved access to palliative care across Canada. The CNA was therefore pleased to see its recommendations reflected in *Bill C‐14*, which legalized MAiD; the Bill recognized the important role of nursing, both when nurses serve as primary care providers, as in the case of NPs, and when they are part of an interdisciplinary healthcare team. It is interesting to note that the American Nurses Association (2013) weighed in similarly on their physician‐assisted death laws although, in that case, the Association took a strong stand against nursing involvement in assisted dying. This strong involvement of nurses and the nursing profession in assisted dying laws is evidence that nursing is becoming more influential in the construction of law and policy.

In the years since its passing, *Bill C‐14* has remained unchanged. However, in that time, many questions have been raised about whether various clinical presentations make one eligible, or not, for MAiD and whether the law, in its current form, truly reflects the morals and ethics of Canadian society and Canadian healthcare professions. It is also interesting to ask whether a law on such a complex issue can truly reflect a “common” ethic or morality when Canadian society itself is so diverse and when a common Canadian value is to guard that diversity. According to a past decision of the SCC, a “truly free society is one which can accommodate a wide variety of beliefs, diversity of tastes and pursuits, customs and codes of conduct” (R. v. Big M Drug Mart, 1985 at para 94). Hence, MAiD in Canada has engendered numerous ethical and moral issues for nursing practice, not the least of which is how to honour a variety of ethical perspectives in the response to a morally contentious healthcare act that is now embedded in law.

To tease out this interplay of law and morality/ethics, we are going to engage in a hypothetical dialogue, guided by an interviewer, between a participant who speaks to the legal implications of MAiD (Lustitia) and a participant who speaks to its moral and ethical implications (Sophia). The substance of their conversation is, of course, nursing's and nurses’ relationships to MAiD from a legal and moral/ethical perspective. Our job, if we do it well, is to explore how nurses might think about these domains differently, thus avoiding the pitfall of simply conflating legal and moral/ethical considerations. It is important to point out that to do so we are going to have to adopt somewhat purist positions and thus run the risk of treating legal and moral/ethical considerations as mutually explicit domains. Our intent is not to make such a claim for there is a robust body of writings pertaining to nursing ethics that argue both for and against the ethical permissibility of MAiD (Pesut, Greig, et al., 2019). Rather, our job is to illuminate as many viewpoints as possible for consideration by nurses as they attempt to reflect on their own positions in relation to this new end‐of‐life option. Although we will speak as if the ideas are our own, we have cited sources that expand on these ideas in more detail.

## 5 NURSES’ LEGAL AND ETHICAL OBLIGATIONS REGARDING MAID: A CONVERSATION

**Interviewer**: Thank you for joining me today. I'd like to begin by asking a question about the implications of legalization. My understanding is that, in passing legislation related to MAiD, Canada chose a somewhat different approach than in other countries where it has been decriminalized but not legislated. Can you explain the implications of that for me?

**Lustitia**: From a legal perspective, there are five different approaches that a country can take in regard to the issue of assisted death (Luzon, 2019), assuming of course that assisted death remains an ethically contentious act. These range on a continuum from a “status quo” approach in which there are no laws, regulations or guidelines regarding assisted suicide through to either a decriminalization approach or to an approach that involves complete legalization of the process. Legislation is considered to be a “hard”, rather than “soft”, approach to assisted death because it requires a high degree of delegation, obligation and precision (Luzon, 2019). A legislated approach outlines rules and safeguards that specify eligibility, processes through which an assisted death must occur, documentation standards, and oversight by a designated body. In contrast, if a country decriminalizes but does not legislate the act, then the formal rules and obligations associated with the procedure are likely to be less detailed and comprehensive.

In Canada, the legal situation pertaining to MAiD has often been contrasted with the decriminalization of abortion many years ago. Both of these issues speak to the question of autonomy and who owns one's body (Abortion Rights Coalition of Canada, 2018). Abortion was decriminalized in the 1988 SCC *Morgentaler* decision but successive governments have consistently chosen not to legislate it. In contrast, the federal government chose to legislate MAiD even though they had the option available not to do so. One of the reasons why the SCC, in the *Carter* decision, chose to suspend the declaration of invalidity of the *Criminal Code* prohibition on physician‐assisted suicide for 12 months was to allow the federal Parliament to have time to decide upon and craft a legislative response. However, the SCC explicitly recognized in the *Carter* decision that provincial legislatures could also choose to respond to the decision with their own legislation, and provincial regulatory bodies could also elect to formalize their regulatory response.

This distinction between federal and provincial/territorial jurisdiction is important to remember because Canada has a fairly unique healthcare system; even though health care is mandated federally under the *Canada Health Act*, and the assisted suicide prohibition is contained within the federal *Criminal Code* statute, the responsibility for those managing, organizing and delivering healthcare services rests with the provinces and territories. It has therefore remained critical for provinces and territories to ensure that the rights of both patients and healthcare providers under the *Charter* are protected. From a nursing perspective, the legislated approach that was selected meant that regulatory bodies such as professional colleges and health regions would also take an active role in creating guidelines and policies for the practice of registered nurses and NPs. Indeed, one only needs to look at the websites of regulatory bodies and health regions in the various provinces and territories to realize that extensive documentation to govern nursing practice specific to MAiD has been developed.

**Sophia**: I think it is important to note that this legislated approach has also had very real implications for nurses who choose not to participate in MAiD. While Bill C‐14 confirmed that no healthcare provider can be compelled to participate in a MAiD procedure, it was left to provinces, territories and regulatory bodies to decide how conscientious objections to MAiD in their jurisdictions would be handled. One commonality across jurisdictions was that any nurse who claimed a conscientious objection to participating in MAiD should notify their employer in advance of their objection so that nursing leaders could delegate MAiD‐related responsibilities to another healthcare provider (CNA, 2017b). While this certainly seems to be a reasonable requirement, preliminary evidence from across the country suggests that there is actually great variability in how much MAiD‐related care nurse leaders expect from conscientiously objecting nurses (Pesut, Thorne, Stager, et al., 2019). In some jurisdictions, policies dictate that nurses can be reassigned to other patients or even take the day off without pay if MAiD will be occurring on their unit. In other jurisdictions however, policies dictate that nurses must provide all care except that which is directly related to the act of MAiD (i.e. the time of providing medications to end life). In the latter situation, nurses who are conscientious objectors would only have the right to step out of the room during medication administration. They would still be required to stay with the patient and family through all other aspects of care. You can appreciate the effect that these different approaches might have on nurses’ mental and emotional well‐being if they conscientiously object to participating in MAiD.

I would further argue that the legislated approach has exacerbated these difficulties because of the way that it has embedded MAiD within the healthcare system. As an embedded act, it can potentially become a part of every nurse's practice whether they work in hospitals, homecare or residential care. This means that nurses who conscientiously object may find it harder to choose an area of practice where they can be confident that MAiD procedures will occur only infrequently and where it will therefore be relatively easy for them to decline participation. If MAiD instead becomes a regular part of their job, objecting nurses must then ask their colleagues to take over care more often than might be comfortable; such an “ask” could be viewed as placing an additional burden upon colleagues in already busy healthcare climate.

In some jurisdictions, MAiD responsibilities have been integrated into the job descriptions of palliative care nurses. In such cases, nurses who conscientiously object may need to make difficult choices about palliative care as a career choice. From my perspective, when an act is decriminalized but not legislated you tend to find it provided in pockets of practice, similar to what has happened with abortion. But when it is integrated into the healthcare system, it can be more difficult for nurses who do not want to take part.

**Lustitia**: I think you are talking about two very different issues. In the case of abortion, you are talking about typically healthy women who can seek out the appropriate pocket of practice to get the care they need. But that is not the case with MAiD. With MAiD, you have a very ill population who should not have to go somewhere else for treatment. MAiD should be accessible wherever a person is receiving care, whether that be in their home or in an institutional care environment. Even though it is a generally accepted principle that healthcare providers should be able to decline participation in care to which they hold a moral or religious objection, I am still concerned about the impact of such withdrawal on accessibility, especially when we have a landscape in Canada that contains many rural and remote communities. What happens when you have a whole unit of nurses who choose not to be involved with MAiD? This is not unheard of in some palliative care units, the very units where these patients are likely to be receiving care. What if, as is the case in a rural or remote community, there are only a few nurses available to staff such units and those few nurses claim conscientious objection to MAiD? What implications would this have for a patient who wants to access this legally permissible, publicly funded healthcare act? If it means transferring the patient out of their home community, and into another community with non‐objecting healthcare providers, that in itself raises multiple ethical issues about simultaneously removing them from their family, friends and support systems precisely when they are most in need of those supports.

**Interviewer**: This is actually a bit confusing to me. I don't really understand the various obligations of decriminalization versus legalization?

**Lustitia**: When an act such as physician‐assisted suicide is decriminalized, the legal prohibition against it is removed and so the threat of legal sanctions for engaging in that act is no longer present. If Canada had chosen decriminalization over legalizing this act, it would have suggested a view that this particular act is best regulated or addressed by something other than the legal system (Downie, 2004). Decriminalization would have focused MAiD debates primarily on the way that individual cases of MAiD would be carried out and would have relied more (although not necessarily exclusively) on professional standards and already established areas of law, such as negligence law, to provide safeguards for patients. It would not demonstrate, as the passing of legislation does, a consensus position of Canadian society about assisted suicide, when it should be available to patients and how it must be regulated (Sawyer, Williams, & Lowy, 1993). Once an act is enshrined as legal within Canadian law, it becomes something that a person is entitled to access provided they meet the eligibility criteria set out in the legislation. This creates obligations and responsibilities for all those who play a role in the MAiD process, from society generally and government bodies to healthcare regulators, individual practitioners and patients. Some of the difficulties we have encountered since MAiD was legalized, however, have involved finding the appropriate balance between (1) ensuring adequate accessibility to an act that society has collectively determined should be legal; and (2) ensuring that the ethics of individual healthcare practitioners are still respected.

**Sophia**: This positioning of accessibility to MAiD as a human right, backed by legislation, has been impactful on nurses’ roles in health care. In health care, we talk a lot about human rights. We have seen many declarations about the right to palliative care (Brennan, 2007; [Link]) and the right to adequate pain management (Brennan, Lohman, & Gwyther, 2019). However, such declarations typically have little ability to create meaningful change. They are instead ideals to which we aspire. But in the case of MAiD, that right of accessibility now has leverage because it has been enshrined into law and is regulated largely at the provincial and territorial levels through health regions and regulatory agencies. So, whereas decriminalization would most likely have resulted in individualized practices of MAiD, usually through a primary care physician, the over‐riding concern now is to have this available to everyone, similar to other healthcare services, regardless of where they live or receive care (Ireland, 2018; Willick, 2018). Nurses, to varying degrees and because of their status as health region employees, then become involved in this publicly funded, healthcare service.

This emphasis on accessibility resulted in the establishment of MAiD teams and coordination services in some provinces, funded by health regions, so that patients would not be required to go through their primary physician to request MAiD. This meant that MAiD became embedded within health care, but not necessarily within the relationships that have traditionally defined an individual's health care. For example, individuals in Canada can request and receive MAiD from healthcare providers with whom they have had no previous relationship, and they can do so without any input from their family physician or from other healthcare providers already providing care to that person (e.g. specialists, homecare nursing). You can imagine then that this embeddedness within publicly funded services will have ramifications for those who conscientiously object, particularly if that objection affects accessibility. For example, some NPs in rural or remote locales may feel obligated to engage in the MAiD process if they are the only eligible assessors or providers in the area (Schiller, 2017). 

**Interviewer**: That is an interesting point. I understand that this particular debate of accessibility is leading to some difficult decisions around publicly funded, faith‐informed institutions.

**Lustitia**: In the Carter case, the SCC stated that their decision to strike down the Criminal Code prohibition on physician‐assisted dying would not compel physicians to provide this procedure to patients. They recognized that a decision of whether or not to be part of an assisted death would be a matter of a physician's own individual conscience and/or religious belief and so governments (federal and provincial) and healthcare regulatory bodies would need to determine the best way to reconcile physician and patient needs in this regard. Similarly, Bill C‐14 states that “everyone has freedom of conscience and religion [emphasis added]” (preamble) and “nothing in this Act affects the guarantee of freedom of conscience and religion” (preamble) and that nothing in the law “compels an individual to provide or assist in providing medical assistance in dying [emphasis added]” (s. 241.2(9)). Neither the Carter decision nor Bill C‐14 specifically address whether an institution can prevent MAiD from occurring on its premises because of the religious beliefs espoused by that institution, rather than the beliefs of an individual practitioner. This has been an ongoing point of contention since the passage of Bill C‐14 because many Catholic healthcare institutions in Canada have prevented MAiD from taking place on their premises, sometimes to the point of preventing even assessments from occurring there, despite the fact that they are publicly funded institutions.

It is important to note that publicly funded faith‐informed health care in Canadian provinces and territories is typically subject to an agreement or memorandum of understanding between the province/territory and the provider institution. These documents usually allow the faith‐informed providers to conduct their own affairs and carry out their religious missions, thereby allowing them to restrict or prevent the provision of those services that conflict with their religious teachings (De Bono, 2017). While many such institutions will facilitate the transfer of MAiD‐seeking patients to non‐objecting facilities, one needs to remember that such patients are typically at end‐of‐life and suffering immensely; this, of course, raises issues about the ethics of physically moving these patients to new facilities, requiring them to quickly develop therapeutic relationships with new healthcare providers, and needing us to consider whether a patient's personal support people are financially and physically able to follow them to their new location.

Of course, one bigger question is: can “bricks and mortar” truly conscientiously object? In other words, what happens when we are not dealing with the conscientious objection of an individual clinical provider but rather the claimed objection of an institution as a whole? It is tempting to say “well, just don't seek care at that institution if you want to access MAiD”. However, not everyone chooses where they will receive health care (e.g. if an ambulance needs to take you to the nearest facility) and not everyone has multiple healthcare institutions in their community to choose from. We certainly have not yet resolved this issue; although, we do anticipate that we will see this issue of conscientiously objecting facilities challenged through the courts over the next while.

**Sophia**: I have watched this debate unfold with some concern as the implications seem quite profound. I think about the possibility that some major healthcare organizations that currently offer important healthcare services to Canadians may lose public funding because they refuse to allow MAiD on‐site. This would effectively close their facilities. Faith‐informed health care remains a major contributor to the public healthcare system, and I am not sure how we would easily replace those important contributions. Although this seems like a pragmatic consideration, closing down major healthcare providers would quickly become a moral issue because of the effect on accessibility. The other alternative would be for these institutions to change their stance in relation to MAiD, certainly not an easy thing to ask when there have often been centuries of theological debate and thinking to inform their position on this matter. Further, the loss of faith‐informed health care would represent the loss of a unique cultural identity in Canada, an identity that may think differently about healthcare‐related concepts such as choice, dignity, and suffering (Beaman & Steele, 2018). I can't help but think back to the idea that a truly free and healthy society will allow for a range of ethical perspectives. I have been struck by arguments claiming that it is cruel to transfer patients at their end‐of‐life to other institutions that permit MAiD at the same time as we regularly transfer patients from hospital to hospice beds (even in different geographic locations) to ensure they receive the care they need. Further, the concern that Lustitia raises about requiring patients to develop new relationships of care upon transfer may be less relevant if those providing MAiD are individuals who have no previous relationship of care anyway.

I believe we need to carefully consider both accessibility for patients and the well‐being of healthcare providers, and in particular, support for their moral convictions (Canadian Medical Protective Association, 2019). In the debate about accessibility, we may forget that there are actually two parties involved in MAiD: those who request it and those who have the responsibility for administering it. The rights of both of these parties are enshrined in the *Charter* and *Bill C‐14*. Nurses in our study told us that their comfort level with MAiD is often inversely proportional to their responsibility for the act. In other words, they generally feel okay about it unless they are the ones who would be responsible for providing it. This dynamic is important for us to understand and it really leads to the question of what constitutes support if one is not willing to engage with the act. We also know that assisting with, or providing, MAiD is deeply impactful, both positively and negatively. Therefore, in creating workspaces that integrate “regular” nursing roles (such as palliative care) with MAiD, we run the risk of placing nurses in ethically distressing situations. In the face of legalization, and the resulting discourse of accessibility, we need to be doubly sure that we create spaces in which nurses can feel free to live out their most deeply held values. This concern for being able to live out these deeply held values would apply just as much to those conscientious providers who believe that MAiD is a compassionate and necessary response to suffering. How can we best support those providers who are personally willing to participate in MAiD but work within institutions where MAiD is not allowed?

**Interviewer**: These sound like difficult decisions for nurses to make. How can nurses go about making these decisions and what factors do nurses need to consider before they become involved in MAiD? 

**Lustitia**: Legally, nurses need to make sure that they are knowledgeable and competent with respect to their role in MAiD. Generally, regulatory agencies responsible for nursing practice have acknowledged that this is not an entry to practice competency and requires additional education (Pesut, Thorne, Stager, et al., 2019). Further, nurses need to know and understand the various policies that impact their participation and practice, including those from regulatory agencies, government and employers. Where any discrepancy between these policies exists, nurses will be required to follow those that are most restrictive. This means that, in some jurisdictions within Canada, NPs are not allowed to act as assessors and providers, even though Bill C‐14 allows them to do so. What is important to note is that registered nurses will also play important roles in fielding initial MAiD requests, organizing care, and providing supports for patients, family, and other healthcare providers and so they too need to be knowledgeable about the legislation and regulations.

**Sophia**: Beyond the regulatory requirements, nurses are also required to follow the responsibilities contained within the CNA (2017a) Code of Ethics for Registered Nurses. The most central responsibilities related to MAiD are to declare oneself as a conscientious objector if that is the case and then to ensure that patients are never abandoned as a result of this objection. However, preliminary evidence from nurses in Canada suggests that this process is a bit more complicated than it might initially appear. Nurses may not necessarily be confident in their initial moral positioning but rather need to work it out over time (Beuthin, Bruce, & Scaia, 2018). This shouldn't surprise us when we realize that much of the willingness to participate in MAiD is related to family and peer influences (Lavoie et al., 2016) and, as society becomes more familiar with MAiD and the views of such influencers evolve, nurses’ moral comfort with the act may evolve over time as well. But what is most important is that nurses do take the time to reflect on their involvement and a number of resources have been developed to help them (CNA, 2017b; Roussel, Beaveridge, & CNA, 2017). We have further learned through our study that actually experiencing a MAiD death is an important step in determining one's level of comfort with future MAiD involvement, and comfort levels may change over time as one encounters situations characterized by different levels of moral complexity. Patients who seem unsure about their decision or who are afraid, family members who disagree or feel traumatized, or deaths in which patients may appear relatively well add a layer of complexity that nurses must grapple with as they determine their comfort level with the procedure.

**Interviewer**: Does this intervention change nurses’ relationships to patients?

**Lustitia**: I would argue that it should not be viewed as changing that relationship. Nursing has always aspired to patient‐centred care and alleviating suffering to the greatest extent possible. Once a patient makes an informed and autonomous decision to receive MAiD, then nurses become a means through which to realize that patient‐centred choice. In that sense, MAiD situations show similarity to those clinical situations where a patient refuses a potentially life‐saving treatment with the understanding that they will die without it. It is not the nurse's decision—the decision belongs to the patient—and the nurse must respect that even if he or she would have chosen differently. Indeed, nurses have suggested that, with MAiD, they now perceive themselves as actually being in a position to definitively relieve irremediable suffering of their patients (Bruce & Beuthin, 2019), something that is not always possible even with the best of palliative care.

**Sophia**: On this question, I think we have some fundamental disagreement. I would argue that the very nature of MAiD cannot help but push new issues to the fore. Yes, NPs are now in a position to definitively end suffering but the means to accomplish that also definitively brings an end to life and, hence, an end to the nurse–patient relationship. Don't get me wrong. I am not claiming that this is an immoral act, I am simply saying that the authority to effectively end a life necessarily brings new dimensions to the nurse–patient relationship. I am not sure that we have fully understood yet what all those new dimensions are, but I do think they warrant further reflection.

**Interviewer**: But does the nurse really have this authority or is he or she simply acting out the requirements of the legislation? It would seem to me that if the eligibility criteria and safeguards are clear, then the nurse can be viewed as simply carrying out a legal healthcare act in the same way as the nurse would perform any other legal healthcare act.

**Lustitia**: I think that the point you are raising is a critical one, and you have worded the qualifier exactly right, i.e. if the requirements and safeguards are clear. An ongoing point of contention with Bill C‐14 is that some of the eligibility criteria are not entirely clear and unambiguous. This is an issue because we, quite reasonably, expect our laws to be clear, and it is certainly reasonable to expect that clinicians will not have to take their best guess at the true meaning of eligibility criteria, particularly for a procedure like MAiD. Many of the discussions about Bill C‐14, from the time that clinicians, lawyers and the general public became aware of proposed language for the statute, centred on the meaning of the phrase “reasonably foreseeable”. One of the eligibility criteria for MAiD in Bill C‐14 is that the patient must be suffering from a “grievous and irremediable medical condition” and the statute tells us the meaning of that phrase, a component of which is that the patient's natural death must have become “reasonably foreseeable”. It was evident from the beginning that there was no clear consensus on what that phrase meant, clinically or legally. Many clinicians took the position that the patient's death needed to be terminal (a position refuted by the government that drafted the legislation), while others felt that death needed to be projected to occur within certain numbers of days or weeks, while still others reviewed actuarial tables to determine the estimated number of months or years left for the patient (given their age, condition, and suffering) and then compared that number to patient conditions in decided legal cases (Martin, 2018). Given that court cases are decided on a highly fact‐specific basis, and most judges resist making sweeping pronouncements in their decisions or stating what they would have decided in different, hypothetical fact scenarios, it is not always easy for clinicians and lawyers to look at past cases and come to a firm conclusion about what a judge would decide in their particular case and for their particular facts. It is a fairly patchwork‐style way of building the law and some of that might have been avoided with clearer statute language.

**Sophia**: I think what we have learned over the past three years, both through practice and court challenges, is that these concepts rely essentially upon the judgement of individual clinicians (Downie & Chandler, 2018). The courts have been clear that the time left until death is not a significant consideration in whether someone is eligible for MAiD but many clinicians still interpret the legal language as requiring such a determination. It therefore lies with clinicians to determine whether the clinical trajectory and presentation makes a person eligible for MAiD. We also know that the criterion of reasonably foreseeable death remains a topic of debate among clinicians, and that clinicians will vary in their opinions as to when someone becomes eligible (Downie & Scallion, 2018). So, as it relates to the criterion of reasonably foreseeable death, clinician judgement becomes paramount. But, it is also important to note that there should be virtually no clinical judgement required for one of the other criteria of “grievous and irremediable medical condition”, which is that the patient must be experiencing intolerable suffering. Suffering can only be defined as what the patient says it is.

What I am suggesting then is that the clinical judgement required to determine MAiD eligibility must also be an ethical judgement, at least as it relates to clinicians’ willingness to provide the act. Because clinicians have so much leeway under the legislation, they must grapple with what decisions they too can live with (Petropanagos, 2019). For example, a patient in the last few days of a terminal illness and a patient newly diagnosed with a life‐limiting neurological condition might both be legally eligible for MAiD. However, the time left to each of these individuals might vary from days to years. Healthcare providers need to work through their moral comfort with these varying trajectories. One of the ways that they come to their decision is to compare this act with other healthcare acts that they have engaged in throughout their careers (2019). Providing MAiD at the very end‐of‐life to relieve what is clearly evident suffering might seem morally different than providing MAiD to someone who still appears to the outside world to have the capacity for quality of life.

I keep coming back to this idea that there are two ethical actors in this healthcare act, and we need to consider them both. Patients may indeed exercise their autonomy under the law, but that autonomy should not be at the cost of ethical injury to another. I think back to a story told by one of the NPs in our study. As she was preparing to provide MAiD, the patient asked whether her maker would judge her for choosing to receive MAiD. The nurse replied, “You? What about me? I am giving the medication!” Although together they had a chuckle about this, the nurse reflected that this question was something that she too had seriously considered. Within the breadth of clinical judgement under the law, nurses must find comfort with their ethical judgement. This should not be misconstrued as ethical judgement of the patient, only their judgement as a competent ethical actor taking responsibility for the provision of MAiD.

**Interviewer**: On that note, I think we will bring this discussion to a close. My thanks to you both for helping us to better understand some of the legal, moral, and ethical nuances of nursing care in Canada in the context of MAiD.

## 6 CONCLUSION

In this paper, we have argued that the situation for Canadian nurses and NPs may be somewhat unique in the international context. With full support of their national advocacy body, the CNA, NPs can independently act as assessors and providers and registered nurses can provide important wrap‐around care during the process. The decriminalization of assisted death that occurred via the *Carter* decision, and the subsequent legalization of MAiD through *Bill C‐14*, was the result of shifting societal beliefs around the value and weight of autonomy and independence in the healthcare context. However, there remains great diversity in how Canadians think about MAiD, ranging from a compassionate intervention to a morally repugnant act.

Despite this spectrum of opinion, the passing of legislation in regard to MAiD has resulted in a high degree of obligation to provide accessibility to this procedure, a high degree of precision in how it is enacted and documented, and the delegation of responsibilities to numerous health policy and decision makers across the country. While MAiD could have been provided outside of the healthcare system (e.g. as is the case in Switzerland) or could have remained primarily the responsibility of individual physicians (e.g. as is the case in Belgium), it has to varying degrees become embedded within healthcare systems in Canada that place a strong emphasis on patient accessibility. This has resulted in the establishment of coordination services and teams, where MAiD assessors and providers find themselves administering assisted death to persons to whom they might otherwise have no relationship of care. It has also resulted in MAiD being integrated into other services, such as palliative care, where it may be difficult for nurses who are conscientious objectors to provide holistic continuity of care.

Although a legislated approach to assisted death may have benefits (e.g. accountability and accessibility), there are undoubtedly challenges associated with it as well. The greatest challenge we see is that legislation, when layered onto the liberal political tendencies to conflate law, morals and policy, tends to suppress the abilities of healthcare providers to remain ethical actors. Further, the fact that the balancing of *Charter* rights of both healthcare providers and patients is actually playing out at the health region policy level means that there is great variability in the way this is approached. Nurses employed by health regions will necessarily be affected by this variability. Those physicians and NPs who run independent practices (and hence, are not really employees of the health system) still have the ability to make independent decisions in relationship to MAiD, although even this is changing. The Ontario Court of Appeal, in a recent unanimous decision, upheld a requirement of the College of Physicians and Surgeons of Ontario that any physician who conscientiously objects to MAiD must provide an effective referral to a non‐objecting, available and accessible physician, NP or agency should the objecting physician receive a MAiD request from a patient (Canadian Medical Protective Association, 2019; Christian Medical & Dental Society of Canada v. College of Physicians & Surgeons of Ontario, 2019). Further, there are other groups lobbying in Canada for physicians to be obligated to inform their clients of the possibility of MAiD, alongside other end‐of‐life options such as palliative care (Daws et al., 2019). Clearly, these movements place limitations on the abilities of conscientiously objecting healthcare providers to fully disengage from what they consider an immoral act.

Legislating a morally contentious act brings to the forefront the question of how we want to be as a society. Is there benefit to creating structures and systems that nudge (to varying degrees) others towards liberal ideals of autonomy, choice and independence? Are we in fact doing a good thing by placing those who see the world differently in positions where they are most likely to conform to our ideals? Or, is there something inherently good, inherently free, about allowing others to give primacy to other ideals? Can we create workspaces that focus on making MAiD accessible while still respecting the moral diversity that such an act engenders? These are not trivial questions for nursing. The moral nature of the environments within which nurses do their work will ultimately determine who will be attracted to the profession. Decisions such as these may also have very real implications for our desire to welcome Indigenous and other nurses who represent the diversity of people that Canada enjoys. Helping nurses to think carefully about the interplay of law and morals/ethics will support their abilities to make those decisions towards a preferred future.

## CONFLICT OF INTEREST

BP, MG and CS declare no conflict of interest. JR is employed by the Canadian Nurses Association, a Canadian policy and advocacy body for nursing.
